# Differential Gene Expression in the Liver of the African Lungfish, *Protopterus annectens*, after 6 Months of Aestivation in Air or 1 Day of Arousal from 6 Months of Aestivation

**DOI:** 10.1371/journal.pone.0121224

**Published:** 2015-03-30

**Authors:** Kum C. Hiong, Yuen K. Ip, Wai P. Wong, Shit F. Chew

**Affiliations:** 1 Natural Sciences and Science Education, National Institute of Education, Nanyang Technological University, Singapore, Republic of Singapore; 2 Department of Biological Science, National University of Singapore, Singapore, Republic of Singapore; Karlsruhe Institute of Technology, GERMANY

## Abstract

The African lungfish, *Protopterus annectens*, can undergo aestivation during drought. Aestivation has three phases: induction, maintenance and arousal. The objective of this study was to examine the differential gene expression in the liver of *P*. *annectens* after 6 months (the maintenance phase) of aestivation as compared with the freshwater control, or after 1 day of arousal from 6 months aestivation as compared with 6 months of aestivation using suppression subtractive hybridization. During the maintenance phase of aestivation, the mRNA expression of *argininosuccinate synthetase 1* and *carbamoyl phosphate synthetase III* were up-regulated, indicating an increase in the ornithine-urea cycle capacity to detoxify ammonia to urea. There was also an increase in the expression of *betaine homocysteine-S-transferase 1* which could reduce and prevent the accumulation of hepatic homocysteine. On the other hand, the down-regulation of *superoxide dismutase 1* expression could signify a decrease in ROS production during the maintenance phase of aestivation. In addition, the maintenance phase was marked by decreases in expressions of genes related to blood coagulation, complement fixation and iron and copper metabolism, which could be strategies used to prevent thrombosis and to conserve energy. Unlike the maintenance phase of aestivation, there were increases in expressions of genes related to nitrogen, carbohydrate and lipid metabolism and fatty acid transport after 1 day of arousal from 6 months aestivation. There were also up-regulation in expressions of genes that were involved in the electron transport system and ATP synthesis, indicating a greater demand for metabolic energy during arousal. Overall, our results signify the importance of sustaining a low rate of waste production and conservation of energy store during the maintenance phase, and the dependence on internal energy store for repair and structural modification during the arousal phase, of aestivation in the liver of *P*. *annectens*.

## Introduction

Lungfishes are an archaic group of Sarcopterygian fishes characterized by the possession of a lung opening off the ventral side of the oesophagus. They hold an important position in the evolutionary tree with regard to water-land transition, during which many important physiological and biochemical adaptations occurred (e.g. air-breathing, urea synthesis, redirection of blood flow, heart partitioning). These adaptations supposedly facilitated the migration of fishes to terrestrial environments, leading to the evolution of tetrapods. There are six species of extant lungfishes, four of which (*Protopterus aethiopicus*, *P*. *amphibius*, *P*. *annectens* and *P*. *dolloi*) are found in Africa. African lungfishes are obligate air-breathers; they typically inhabit fringing weedy areas of lakes and rivers where dissolved oxygen levels are low, daytime temperatures are high, and seasonal drying is common. Without limbs to facilitate locomotion on land, lungfishes would have to passively tolerate desiccation, and aestivation could be the only means for survival under desiccation at high temperature. Aestivation involves corporal torpor at high environmental temperature with absolutely no intake of food and water for an extended period. African lungfishes can aestivate in subterranean mud cocoons for ~4 years [1], which could be the longest aestivation period known for vertebrates.

Traditionally, aestivation experiments on African lungfishes were performed either in mud or in cloth bags in the laboratory [2–5]. Chew et al. [6] were the first to achieve induction of aestivation in *P*. *dolloi* in pure mucus cocoons in air inside plastic boxes. Subsequently, it has been confirmed that *P*. *annectens*, *P*. *aethiopicus* [7–11] and *P*. *amphibius* (Y.K.I. and S.F.C, unpublished observation) can also be induced to aestivate in pure mucus cocoons in air. There are three phases of aestivation. During the induction phase in air, the fish detects environmental cues and turn them into some sort of internal signals that would instill the necessary changes at the behavioral, structural, physiological and biochemical levels in preparation of aestivation. It secretes a substantial amount of mucus which turns into a dry cocoon within 6–8 days. Aestivation begins when the fish is completely encased in a dried mucus cocoon, and there is a complete cessation of feeding and locomotor activities. During the maintenance phase, the fish has to preserve the biological structures and sustain a slow rate of waste production to avoid pollution of the internal environment. It can perpetuate to aestivate under such conditions for more than a year. The aestivating lungfish can be aroused from aestivation by the addition of water. Upon arousal, the fish struggles out of the cocoon and swims, albeit sluggishly, to the water surface to gulp air. After arousal, it excretes the accumulated waste products, and feeds for repair and growth. Completion of aestivation occurs only if arousal is successful; if not, the animal have had apparently succumbed to certain factors during the maintenance phase. Feeding begins approximately 7–10 days after arousal, and the fish grow and develop as normal thereafter.

It is apparent that adaptive (physiological, biochemical and molecular) changes in various organs of the aestivating African lungfish would vary during the three phases of aestivation. However, the majority of studies in the past focused only on the maintenance phase, and there is a dearth of information on the induction and arousal phases of aestivation [12]. Loong et al. [13] pioneered in using suppression subtractive hybridization (SSH) polymerase chain reaction (PCR) to identify aestivation-specific gene clusters in the liver of *P*. *annectens* after 6 days (induction phase) of aestivation in a mucus cocoon in air (normoxia). They reported up- or down-regulation of several gene clusters which were involved in urea synthesis, prevention of clot formation, activation of the lectin pathway for complement activation, conservation of minerals (e.g. iron and copper) and increased production of hemoglobin beta. Since there were up- and down-regulation of mRNA expressions of genes related to ribosomal proteins and translational elongation factors, there could be simultaneous increases in protein degradation and protein synthesis during 6 days of aestivation, confirming the importance of reconstruction of protein structures in preparation for the maintenance phase of aestivation [13].

The liver is involved in diverse metabolic activities which include detoxification, oxidative defense, urea synthesis, carbohydrate and amino acid metabolism, and iron and copper metabolism. Even during the maintenance phase of aestivation, the liver has to continue functioning to detoxify ammonia to urea; only then, would the aestivating fish be able to mobilize protein and amino acid as an energy source for survival during the aestivation process. Therefore, in this study, we continued to examine the effects of 6 months of aestivation and 1 day arousal from 6 months of aestivation on the up- and down-regulation of genes in the liver of *P*. *annectens* using SSH PCR. SSH involves two types of cDNAs: testers (with treatment) and drivers (control). In order to examine differential gene expression in the liver during the maintenance phase (6 months) of aestivation (tester), liver of fish kept in fresh water was used as the driver. Results obtained would indicate changes in gene expression in aestivating fish with reference to non-aestivating fish. However, in order to examine differential gene expression in the liver during the arousal phase (1 day arousal from 6 months of aestivation) of aestivation (tester), liver of fish that had undergone 6 months of aestivation in air were used as driver instead. In this way, results obtained would reveal changes in gene expression in aroused fish with reference to aestivating fish.

The zebrafish nomenclature system (see https://wiki.zfin.org/display/general/ZFIN+Zebrafish+Nomenclature+Guidelines) for genes and proteins of fish origin and the human nomenclature (see http://www.genenames.org/guidelines.html) for genes and proteins of mammalian origin were adopted in this paper. Specifically, for fishes, gene symbols are italicized, all in lower case, and protein designations are the same as the gene symbol, but not italicized with the first letter in upper case.

## Materials and Methods

### Collection and maintenance of fish

*Protopterus annectens* (80–120 g body mass) were imported from Central Africa through a local fish farm in Singapore. They were maintained in plastic aquaria filled with dechlorinated freshwater at pH 7.0 and at 25°C in the laboratory. Water was changed daily. No attempt was made to separate the sexes. Fish were acclimated to laboratory conditions for at least 1 month before experimentation. During the adaptation period, fish were fed with frozen fish meat and food was withheld 96 h prior to experiments.

### Ethics Statement

Approval to undertake this study was obtained from the Institutional Animal Care and Use Committee of the National University of Singapore (IACUC 035/09).

### Experimental conditions and tissue sampling

*Protopterus annectens* were induced to aestivate at 27–29°C and 85–90% humidity individually in plastic tanks (L29 cm x W19 cm x H17.5 cm) containing 15 ml of dechlorinated tap water (adjusted to 0.3‰ with seawater) following the procedure of Chew et al. [6]. During the induction phase of aestivation, the experimental fish would secrete plenty of mucus during the first few days, and the mucus would slowly dry up between day 5 and day 7 to form a mucus cocoon. Aestivation was considered to begin when the fish was fully encased in the cocoon and displayed no locomotor activities. *Protopterus annectens* can be maintained in aestivation for a long period of time and this was regarded as the maintenance phase of aestivation.

Fish maintained in freshwater served as controls. Control fish were killed with an overdose of neutralized MS222 (0.2%) followed with a blow to the head. Aestivating fish were killed on day 186 (6 months; prolonged maintenance phase) or after 1 day arousal from 6 months of aestivation with a blow to the head. The liver was quickly excised and frozen in liquid nitrogen. The frozen samples were kept at-80°C until analysis.

### Total RNA and poly (A) mRNA extraction

Frozen tissues were homogenized using a polytron homogenizer (Kinematica AG, Lucerne, Switzerland) in 400 μl of chaotropic buffer (4.5 M guanidine thiocyanate, 2% N-lauroylsarcosine, 50 mM EDTA (pH 8.0), 25 mM Tris-HCl (pH 7.5), 0.1 M β-mercaptoethanol, 0.2% antifoam A). Total RNA was extracted from the liver, using the chaotropic extraction protocol described by Whitehead and Crawford [14]. The RNA pellet obtained was rinsed twice with 500 μl of 70% ethanol, and further purified using the Qiagen RNeasy Mini Kit (Qiagen Inc., Valencia, CA, USA). The concentration and purity of the purified RNA were determined using the NanoDrop ND-1000 spectrophotometer (Thermo Fisher Scientific Inc., Wilmington, DE, USA). The RNA quality was determined by visualising the presence of the 18S and 28S ribosomal RNA bands using the BioRad Universal Hood II gel documentation system (BioRad, Hercules, CA, USA) after carrying out electrophoresis of 1 μg of RNA on 1% (w/v) agarose gel in TAE buffer (40 mM Tris-acetate, 1 mM EDTA, pH 8.0) with nucleic acid staining dye GelRed (1:20000, Biotium Inc., Hayward, CA, USA) at 100 V for 30 min. The presence of sharp 28S and 18S bands in the proportion of about 2:1 indicate the integrity of the total RNA.

Poly (A) mRNA was extracted from 200 μg of total RNA using the Oligotek mRNA kit (Qiagen Inc.). The RNA sample (200 μg) was mixed with 15 μl of Oligotex suspension (resin) and was heated at 70°C for 3 min and then cooled at 25°C for 10 min. The Oligotex:mRNA complex was spun at 14,000 x*g* and the pellet obtained was resuspended in 400 μl of Buffer OW2 (Qiagen Inc.) and then passed through a small spin column by centrifuging at 14,000 x*g* for 1 min. The column was washed with another 400 μl of Buffer OW2. The resin in the column was resuspended with 50 μl of hot (70°C) Buffer OEB (Qiagen Inc.) and eluted by centrifugation at 14,000 x*g* for 1 min to obtain the Poly (A) RNA. Another 50 μl of hot (70°C) Buffer OEB was added to the column and the process was repeated to ensure maximal Poly (A) mRNA yield.

### Construction of SSH libraries

Two sets of forward (up-regulated genes) and reverse (down-regulated genes) SSH libraries for the liver were generated using the PCR-Select cDNA subtraction kit (Clontech Laboratories, Inc., Mountain View, CA, USA); one set for fish aestivated for 6 months in air (prolonged maintenance phase) with reference to the freshwater control, and the other set for fish that was aroused for 1 day after 6 months of aestivation in air (arousal phase) with reference to 6 months of aestivation in air. Two micrograms of poly (A) mRNA from each condition was used for cDNA synthesis. After the first and second strand synthesis, the double stranded cDNA from both groups were digested with Rsa I. A portion of the digested cDNA was ligated with either Adapter 1 or Adaptor 2R, and the rest was saved for subsequent usage as the driver for hybridization. The forward library was generated from the hybridization between adapter-ligated cDNA obtained from fish that had undergone 6 months of aestivation in air or fish that were recovered for 1 day (tester) and Rsa I-digested cDNA from the control fish kept in fresh water or fish aestivated for 6 months in air (driver). The reverse library was made the same way, except that the adapter-ligated cDNA from the control in fresh water or 6 months of aestivation served as the tester while the Rsa I-digested cDNA from fish aestivated for 6 months in air or fish that were recovered for 1 day acted as the driver, respectively. The driver cDNA was added in excess to remove common cDNA by hybrid selection, leaving over-expressed and novel tester cDNAs to be recovered and cloned. The PCR amplification of the differentially expressed cDNAs was performed using the Advantage cDNA polymerase mix (Clontech Laboratories, Inc.) and 9902 Applied Biosystems PCR thermal cycler (Life Technologies Corporation, Carlsbad, CA, USA). The primary and secondary PCR amplification of these reciprocal subtractions of cDNA from the control and aestivated fish produced 1 forward and 1 reverse SSH libraries enriched in differentially expressed transcripts.

Differentially expressed cDNAs were cloned using pGEM-T easy vector system kit (Promega Corporation, Madison, WI, USA), transformed into chemically competent JM109 *Escherichia coli* (Promega Corporation), and plated onto Luria-Bertani (LB) agar with ampicillin, 5-bromo-4-chloro-3-indolyl-β-D-galactopyranoside (X-gal) and isopropyl β-D-thiogalactopyranoside (IPTG). Selected white colonies were grown overnight in LB broth with ampicillin. The plasmids were extracted using the resin-based plasmid miniprep kit (Axygen Biosciences, Union City, CA, USA). The plasmids were quantified by the NanoDrop ND-1000 spectrophotometer. Approximately 80–100 ng of plasmid DNA was used in BigDye Terminator v3.1 Cycle Sequencing Kit (Life Technologies Corporation) with 2 μM T7 primers. Excess fluorescent nucleotides and salts were removed from the samples by ethanol precipitation. The dried samples were resuspended in Hi-Di Formamide (Life Technologies Corporation) before loading to the Prism 3130XL sequencer (Life Technologies Corporation). A total of 500 clones for each forward and reverse library were selected for sequencing.

Sequence output was exported as text and edited manually to remove vector sequences using BioEdit Sequence Alignment Editor software version 7.0.9 [15]. BLAST searches were performed using the tBLASTx algorithm [16] and default search conditions. Proteins were considered significant when the *E* value was <1E-04. The annotated sequences were grouped based on Gene Ontology classification. The sequences were deposited in Genbank EST database and were assigned with accession numbers JZ575382 to JZ575617.

### Relative quantitative real-time PCR (qPCR)

In order to validate the changes obtained in the SSH studies, nine genes were selected for the determination of mRNA expression using quantitative real-time PCR (qPCR). These include *acyl-CoA desaturase* (*acd*), *argininosuccinate synthetase 1* (*ass1*), *betaine-homocysteine S-methyltransferase 1* (*bhmt1*), *ceruloplasmin* (*cp*), *carbamoyl phosphate synthetase III* (*cpsIII*), *fumarate hydratase* (*fh*), *ferritin light chain* (*ftl*), *glyceraldehyde-3-phosphate dehydrogenase* (*gapdh*) and *superoxide dismutase 1* (*sod1*). Prior to first strand cDNA synthesis, RNA from the liver of fish kept in fresh water, aestivated for 6 months in air or aroused for 1 day after 6 months of aestivation in air were treated separately with Deoxyribonuclease I (Qiagen Inc.) to remove any contaminating genomic DNA. First strand cDNA was synthesized from 1 μg of total RNA using random hexamer primer and the RevertAid first stand cDNA synthesis kit, following the manufacturer’s instruction (Thermo Fisher Scientific Inc). mRNA expressions of the selected genes were quantified using a StepOnePlus Real-Time PCR System (Life Technologies Corporation). Each PCR reaction contained 5 μl of 2x Fast SYBR Green Master Mix (Life Technologies Corporation), a certain aliquot of gene-specific primers (listed in Table 1) and 0.1–2 ng of cDNA in a total volume of 10 μl. Samples were run in triplicate. qPCR reactions were performed with the following cycling conditions: 95°C for 20 s (1 cycle), followed by 40 cycles of 95°C for 3 s and 60°C of 30 s. Data was collected at each elongation step. Each run was followed by a melt curve analysis by increasing the temperature from 60°C to 95°C at 0.3°C increment to confirm the presence of only a single PCR product. In addition, random PCR products were electrophoresed in a 1.8% agarose gel to verify that only one band was present. All the data were normalized to the abundance of *β-actin* mRNA. The amplification efficiencies for *β-actin* and all selected genes were between 90–100%. The subsequent application of the 2^-ΔΔCT^ calculation for relative quantification was validated by confirming that the variation between the amplification efficiencies of the target and reference gene through a 100-fold dilution remained relatively constant [17]. The mean fold-change values were transformed into logarithmic values (log_2_) to enable valid statistical analysis.

**Table 1:** Primers used for quantitative real-time PCR on *acyl-CoA desaturase* (*acd*), *argininosuccinate synthetase 1* (*ass1*), *betaine-homocysteine S-methyltransferase 1* (*bhmt1*), *ceruloplasmin* (*cp*), *carbamoyl-phosphate synthetase III* (*cpsIII*), *fumarate hydratase* (*fh*), *ferritin light chain* (*ftl*), *glyceraldehyde-3-phosphate dehydrogenase* (*gapdh*), *superoxide dismutase 1* (*sod1*) from the liver of *Protopterus annectens*.

Gene	Primer sequence (5’ to 3’)
*acd* (JZ575387)	Forward (5’-GTCAGCCACCACAACACA-3’)
	Reverse (5’-ACATCTCCCTGCCCATTCT-3’)
*ass1* (JZ575533)	Forward (5’-CATGGAGTATGGATGCTAACCT-3’)
	Reverse (5’-GTACTGTCTTATCGTTGAGATTGG-3’)
*bhmt1* (JZ575536)	Forward (5’-TGCTTACTTGACTCCTGATTGTG-3’)
	Reverse (5’-CTTGCGTACTTGTGAATATCCCA-3’)
*cp* (JZ575541)	Forward (5’-TGGACACAGCTTTGATTATAAGAG-3’)
	Reverse (5’-CAGTCATTTGTAGTGCTTGGA-3’)
*cpsIII* (JZ575539)	Forward (5’-TTGGTTACCCAGTGATGATCCGA-3’)
	Reverse (5’-CACTTCATACTCCACCTCCTTCC-3’)
*fh* (JZ575565)	Forward (5’-TAGTAACAGCACTCAACCCAC-3’)
	Reverse (5’-GCTTGACCCACTGATCAAACTG-3’)
*ftl* (JZ575418)	Forward (5’-CTCAAATTCCAGAATCGCCGT-3’)
	Reverse (5’-TAGTCCATAGCCTGCATCCCA-3’)
*gapdh* (JZ575429)	Forward (5’-ATGACAACCGTCCATGCT-3’)
	Reverse (5’-AATGACTTTGCCGACTGCC-3’)
*sod1* (JZ575606)	Forward (5’-ATGTAGGTGATCTTGGAAATGTG-3’)
	Reverse (5’-TGCCCAAGTCATCTTCTTTCTC-3’)
*β-actin*	Forward (5’-CATACTGTGCCCATTTATGAAGGT-3’)
	Reverse (5’-CAAGTCACGGCCAGCTAAATC-3’)

### Statistical analysis

Results for qPCR were presented as means ± standard errors of the mean (S.E.M.). Student’s t-test was used to evaluate the difference between means. Differences with *P*<0.05 were regarded as statistically significant.

## Results

### SSH libraries from liver of *P*. *annectens* after 6 months of aestivation (with fresh water control as the driver)

Two SSH-generated libraries, forward (Table 2) and reverse (Table 3), were constructed for genes that were up- and down-regulated, respectively, in the liver of *P*. *annectens* which had undergone 6 months of aestivation in air. A total of 98 genes were identified from these SSH libraries, of which 20 genes were up-regulated (Table 2) and 78 genes were down-regulated (Table 3). There were 340 unidentified sequences which could be genes that are yet to be characterized in *P*. *annectens*. Ribosomal protein S12 appeared in both forward and reverse subtraction libraries, indicating that it could be false positives or encoding for different isoforms of the same protein.

**Table 2:** Known transcripts found in the forward library (up-regulation) obtained by suppression subtractive hybridization PCR from the liver of *Protopterus annectens* aestivated for 6 months in air with fish kept in fresh water as the reference for comparison.

Group and Gene	Gene symbol	*P*. *annectens* accession no.	Homolog species	E-value	No of clones	Biological processes
**Nitrogen metabolism**						
*argininosuccinate synthetase 1*	*ass1*	JZ575533	*Xenopus laevis*	1E-47	3	Arginine biosynthetic process
*carbamoyl-phosphate synthetase III*	*cpsIII*	JZ575539	*Xenopus laevis*	1E-53	18	Glutamine metabolic process
**Amino acid, polyamine and nucleotide metabolism**						
*betaine-homocysteine S-methyltransferase 1*	*bhmt1*	JZ575536	*Xenopus laevis*	8E-95	39	Methionine biosynthetic process
**Tricarboxylic acid cycle**						
*fumarate hydratase*	*fh*	JZ575565	*Danio rerio*	3E-90	19	Tricarboxylic acid cycle
**Cell structure**						
*actin*, *beta*	*actb*	JZ575523	*Cynops ensicauda*	1E-117	44	Cell structure
**Protein synthesis, transport and folding**						
*ribosomal protein L11*	*rpl11*	JZ575583	*Protopterus dolloi*	6E-133	13	Translation
*ribosomal protein L18*	*rpl18*	JZ575584	*Protopterus dolloi*	3E-130	9	Translation
*ribosomal protein L27a*	*rpl27a*	JZ575586	*Xenopus laevis*	9E-22	5	Translation
*ribosomal protein L29*	*rpl29*	JZ575587	*Ictalurus punctatus*	1E-63	29	Translation
*ribosomal protein S12 fragment 1*	*rps12*	JZ575591	*Xenopus laevis*	7E-56	1	Translation
*ribosomal protein S25*	*rps25*	JZ575594	*Xenopus laevis*	5E-31	5	Translation
*ribosomal protein S29*	*rps29*	JZ575595	*Salmo salar*	5E-20	1	Translation
*ribosomal protein S2e*	*rps2e*	JZ575590	*Xenopus laevis*	5E-77	9	Translation
*40S ribosomal protein S2*	*rps2*	JZ575520	*Salmo salar*	1E-72	36	Translation
*60S ribosomal protein L6*	*rpl6*	JZ575521	*Salmo salar*	1E-77	1	Translation
**Transport**						
*serum lectin*	*sln35-a*	JZ575603	*Xenopus laevis*	3E-15	1	Protein transport
*globin Y*	*gby*	JZ575566	*Xenopus laevis*	6E-05	3	Oxygen transport
**Others**						
*DEAD (Asp-Glu-Ala-Asp) box polypeptide 21*	*ddx21*	JZ575554	*Salmo salar*	6E-46	47	Unclassified
*small EDRK-rich factor 2*	*serf2*	JZ575604	*Oncorhynchus mykiss*	1E-15	2	Unclassified
*group-specific component (vitamin D binding protein)*	*gc*	JZ575567	*Xenopus (Silurana) tropicalis*	3E-27	99	Vitamin D metabolic process

**Table 3:** Known transcripts found in the reverse library (down-regulation) obtained by suppression subtractive hybridization PCR from the liver of *Protopterus annectens* aestivated for 6 months in air with fish kept in fresh water as the reference for comparison.

Group and Gene	Gene symbol	*P*. *annectens* accession no.	Homolog species	E-value	No of clones	Biological processes
**Carbohydrate metabolism**						
*fructose-bisphosphate aldolase C*	*aldoc*	JZ575564	*Salmo salar*	4E-09	2	Glycolysis
**Amino acid, polyamine and nucleotide metabolism**						
*inter-alpha trypsin inhibitor*, *heavy chain 2*	*itih2*	JZ575571	*Xenopus laevis*	8E-18	4	Hyaluronan metabolic process
**Blood coagulation**						
*apolipoprotein H*	*apoh*	JZ575532	*Xenopus (Silurana) tropicalis*	8E-35	8	Regulation of blood coagulation
*serine (or cysteine) proteinase inhibitor*, *clade C (antithrombin)*, *member 1*	*serpinc1*	JZ575599	*Xenopus laevis*	3E-17	3	Blood coagulation
*beta-2-glycoprotein 1 precursor*	*b2g1*	JZ575535	*Salmo salar*	2E-20	1	Regulation of blood coagulation
*coagulation factor II precursor*	*f2*	JZ575542	*Xenopus laevis*	3E-36	2	Blood coagulation, platelet activation
*fibrinogen alpha*	*fga*	JZ575561	*Xenopus laevis*	4E-78	3	Blood coagulation, platelet activation
*fibrinogen beta*	*fgb*	JZ575562	*Xenopus laevis*	4E-78	2	Blood coagulation, platelet activation
*fibrinogen gamma*	*fgg*	JZ575563	*Xenopus laevis*	4E-78	2	Blood coagulation, platelet activation
**Complement**						
*CD46 antigen*, *complement regulatory protein*	*cd46*	JZ575540	*Equus caballus*	8E-06	2	complement
*peptidoglycan recognition protein 2*	*pglyrp2*	JZ575577	*Xenopus (Silurana) tropicalis*	3E-23	1	Immune response, peptidoglycan catabolic process
*complement C3 precursor alpha chain fragment 1*	*c3*	JZ575543	*Protopterus aethiopicus*	0	5	Complement activation
*complement C3 precursor alpha chain fragment 2*	*c3*	JZ575544	*Protopterus aethiopicus*	0	4	Complement activation
*complement C3 precursor alpha chain fragment 3*	c3	JZ575545	*Protopterus aethiopicus*	0	2	Complement activation
*complement component receptor 1*	*cr1*	JZ575551	*Canis familiaris*	1E-06	1	Complement activation
*complement component 1*	*c1*	JZ575547	*Xenopus (Silurana) tropicalis*	1E-31	1	Innate immune response
*complement component 4 binding protein*, *alpha*	*c4bpa*	JZ575548	*Macaca mulatta*	3E-15	3	Innate immune response
*complement component 9*	*c9*	JZ575549	*Xenopus (Silurana) tropicalis*	1E-04	1	Innate immune response
*complement component factor h*	*cfh*	JZ575550	*Xenopus laevis*	3E-06	1	Complement activation
*complement C4-2*	*c4b*	JZ575546	*Cyprinus carpio*	5E-08	1	Complement activation
*complement receptor-like protein 1*	*cr1l*	JZ575552	*Oncorhynchus mykiss*	1E-07	1	Complement activation
**Iron, copper metabolism and transport**						
*aminolevulinic acid synthase 1*	*alas1*	JZ575530	*Protopterus dolloi*	1E-127	1	Heme biosynthetic process
*ceruloplasmin*	*cp*	JZ575541	*Danio rerio*	5E-46	33	Copper ion transport
*hemopexin*	*hpx*	JZ575569	*Rattus norvegicus*	7E-17	3	Hemoglobin metabolic process
*transferrin fragment 1*	*tf*	JZ575609	*Xenopus laevis*	3E-16	6	Iron ion transport
*transferrin fragment 2*	*tf*	JZ575610	*Xenopus laevis*	3E-16	8	Iron ion transport
**Protein synthesis, transport and folding**						
*eukaryotic translation elongation factor 2*	*eef2*	JZ575557	*Xenopus (Silurana) tropicalis*	7E-126	1	Translation
*eukaryotic translation initiation factor 5A*	*eif5a*	JZ575558	*Danio rerio*	3E-07	1	Translation
*ribosomal protein L21*	*rpl21*	JZ575585	*Mus musculus*	3E-16	1	Translation
*ribosomal protein L36A*	*rpl36a*	JZ575588	*Danio rerio*	2E-32	1	Translation
*ribosomal protein P2*	*rplp2*	JZ575589	*Ictalurus punctatus*	2E-76	4	Translational elongation
*ribosomal protein S12 fragment 2*	*rps12*	JZ575592	*Xenopus laevis*	4E-41	2	Translation
*ribosomal protein S17*	*rps17*	JZ575593	*Xenopus laevis*	4E-104	2	Translation
*protein AMPB*	*ampb*	JZ575529	*Taeniopygia guttata*	5E-14	2	Protein maturation, transport
*serum albumin*	*alb*	JZ575602	*Ornithorhynchus anatinus*	1E-36	2	Transport
*translation initiation factor eIF4A I*	*eif4a1*	JZ575611	*Xenopus laevis*	6E-83	1	Translation
*alpha 1 microglobulin*	*iti*	JZ575526	*Xenopus (Silurana) tropicalis*	4E-25	2	Protein maturation
*sec61-alpha*	*sec61a*	JZ575598	*Salmo salar*	8E-148	8	Protein transport
**Protein degradation**						
*hyaluronan binding protein 2*	*habp2*	JZ575570	*Danio rerio*	3E-16	2	Proteolysis
**Oxidation reduction**						
*NADH dehydrogenase (ubiquinone) 1 alpha subcomplex 4*	*ndufa4*	JZ575575	*Danio rerio*	5E-24	2	Electron transport chain
*cytochrome P450*, *family 2*, *subfamily D*, *polypeptide 6*	*cyp2d6*	JZ575553	*Xenopus (Silurana) tropicalis*	7E-12	1	Oxidation reduction
*NADPH-P450 reductase*	*por*	JZ575576	*Xenopus laevis*	7E-38	1	Oxidation reduction
**Cell growth, cycle and proliferation**						
*deiodinase type III*	*dio3*	JZ575555	*Neoceratodus forsteri*	3E-121	3	Hormone biosynthetic process, positive regulation of multicellular organism growth
*thymidine kinase 2*	*tk2*	JZ575607	*Homo sapiens*	2E-05	1	Cellular DNA replication
**Transcription**						
*zinc finger*, *CCHC domain containing 8*	*zcchc8*	JZ575617	*Xenopus laevis*	6E-07	1	mRNA processing, RNA splicing
*metastasis associated 1*	*mta1*	JZ575573	*Xenopus (Silurana) tropicalis*	1E-09	1	Regulation of transcription
**Antioxidative stress**						
*superoxide dismutase 1*	*sod1*	JZ575606	*Xenopus (Silurana) tropicalis*	4E-34	2	Response to oxidative stress
*stress-associated endoplasmic reticulum protein 1*	*serp1*	JZ575605	*Xenopus (Silurana) tropicalis*	2E-38	1	Endoplasmic reticulum unfolded protein response, protein transport
**Transport**						
*potassium channel*, *subfamily K*	*kcnk*	JZ575579	*Homo sapiens*	4E-08	1	Potassium ion transport
*transthyretin*	*ttr*	JZ575612	*Xenopus (Silurana) tropicalis*	4E-09	2	Thyroid hormone generation, transport
**Others**						
*ribosomal protein 5S-like protein*	*rna5s*	JZ575582	*Prionace glauca*	5E-71	6	Unclassified
*abhydrolase domain containing 11*	*abhd11*	JZ575522	*Xenopus (Silurana) tropicalis*	1E-10	2	Unclassified
*adaptor-related protein complex 1*, *mu 1*	*ap1m1*	JZ575524	*Danio rerio*	7E-38	2	Vesicle-mediated transport
*alcohol dehydrogenase 3*	*adh3*	JZ575525	*Protopterus dolloi*	2E-76	1	Ethanol catabolic process, retinoic acid metabolic process, oxidation reduction
*alpha-2 macroglobulin fragment 1*	*a2m*	JZ575527	*Danio rerio*	3E-33	10	Female pregnancy
*alpha-2 macroglobulin fragment 2*	*a2m*	JZ575528	*Danio rerio*	3E-33	8	Female pregnancy
*apoferritin higher subunit*	*fth1*	JZ575531	*Rana catesbeiana*	1E-90	1	Unclassified
*beta-2-globin*	*hbb*	JZ575534	*Xenopus laevis*	5E-05	1	Unclassified
*calumenin*	*calu*	JZ575538	*Xenopus (Silurana) tropicalis*	1E-63	1	Unclassified
*endonuclease domain containing 1*	*endod*	JZ575556	*Xenopus laevis*	6E-07	2	Unclassified
*fetuin B fragment 1*	*fetub*	JZ575559	*Xenopus (Silurana) tropicalis*	2E-20	1	Unclassified
*fetuin B fragment 2*	*fetub*	JZ575560	*Xenopus (Silurana) tropicalis*	2E-20	6	Unclassified
*heme-binding protein 2*	*hebp2*	JZ575568	*Danio rerio*	4E-25	2	Unclassified
*kh domain-containing transcription factor B3*	*igf2bp3-b*	JZ575572	*Xenopus laevis*	7E-10	16	Unclassified
*microtubule-associated protein 1 light chain 3 alpha*	*map1lc3a*	JZ575574	*Xenopus laevis*	3E-30	1	Autophagy
*phosphotyrosine interaction domain containing 1*	*pid1*	JZ575578	*Danio rerio*	1E-60	8	Unclassified
*c6orf58 homolog*	*c6orf58*	JZ575537	*Callorhinchus milii*	2E-21	1	Unclassified
*progesterone receptor membrane component 1*	*pgrmc1*	JZ575580	*Xenopus (Silurana) tropicalis*	6E-07	1	Unclassified
*protein GTLF3B*	*natd1*	JZ575581	*Xenopus laevis*	3E-10	4	Unclassified
*run domain-containing protein 1*	*rundc1*	JZ575596	*Salmo salar*	7E-16	3	Unclassified
*saxiphilin*	*sax*	JZ575597	*Rana catesbeiana*	9E-11	24	Unclassified
*serotransferrin B*	*tfb*	JZ575600	*Xenopus laevis*	3E-30	5	Unclassified
*serotransferrin-1*	*tf1*	JZ575601	*Salmo salar*	2E-11	1	Unclassified
*tumor protein*, *translationally-controlled 1*	*tpt1*	JZ575613	*Xenopus laevis*	2E-07	1	Anti-apoptosis
*warm-temperature-acclimation-related-65 kDa-protein-like-protein fragment 1*	*wap65-like*	JZ575614	*Oryzias latipes*	8E-12	12	Unclassified
*warm-temperature-acclimation-related-65 kDa-protein-like-protein fragment 2*	*wap65-like*	JZ575615	*Oryzias latipes*	8E-12	5	Unclassified
*warm-temperature-acclimation-related-65 kDa-protein-like-protein fragment 3*	*wap65-like*	JZ575616	*Oryzias latipes*	8E-12	3	Unclassified
*transducer of ERBB2*, *1b*	*tob1b*	JZ575608	*Danio rerio*	2E-89	2	Unclassified

The forward library indicated the up-regulation of *bhmt1* and *fh* expression levels in the liver of *P*. *annectens* after 6 months of aestivation. Certain genes related to nitrogen metabolism such as *ass1* and *cps III* and a number of ribosomal genes that was involved in protein synthesis were also up-regulated (Table 2).

The reverse library indicated the down-regulation of expression levels of genes related to antioxidative stress (e.g. *sod1*) and copper transport (e.g. *cp*) in the liver of *P*. *annectens* after 6 months of aestivation. The mRNA expression levels of some genes involved in complement activation, blood coagulation and iron transport were also down-regulated (Table 3).

**Fig 1:**
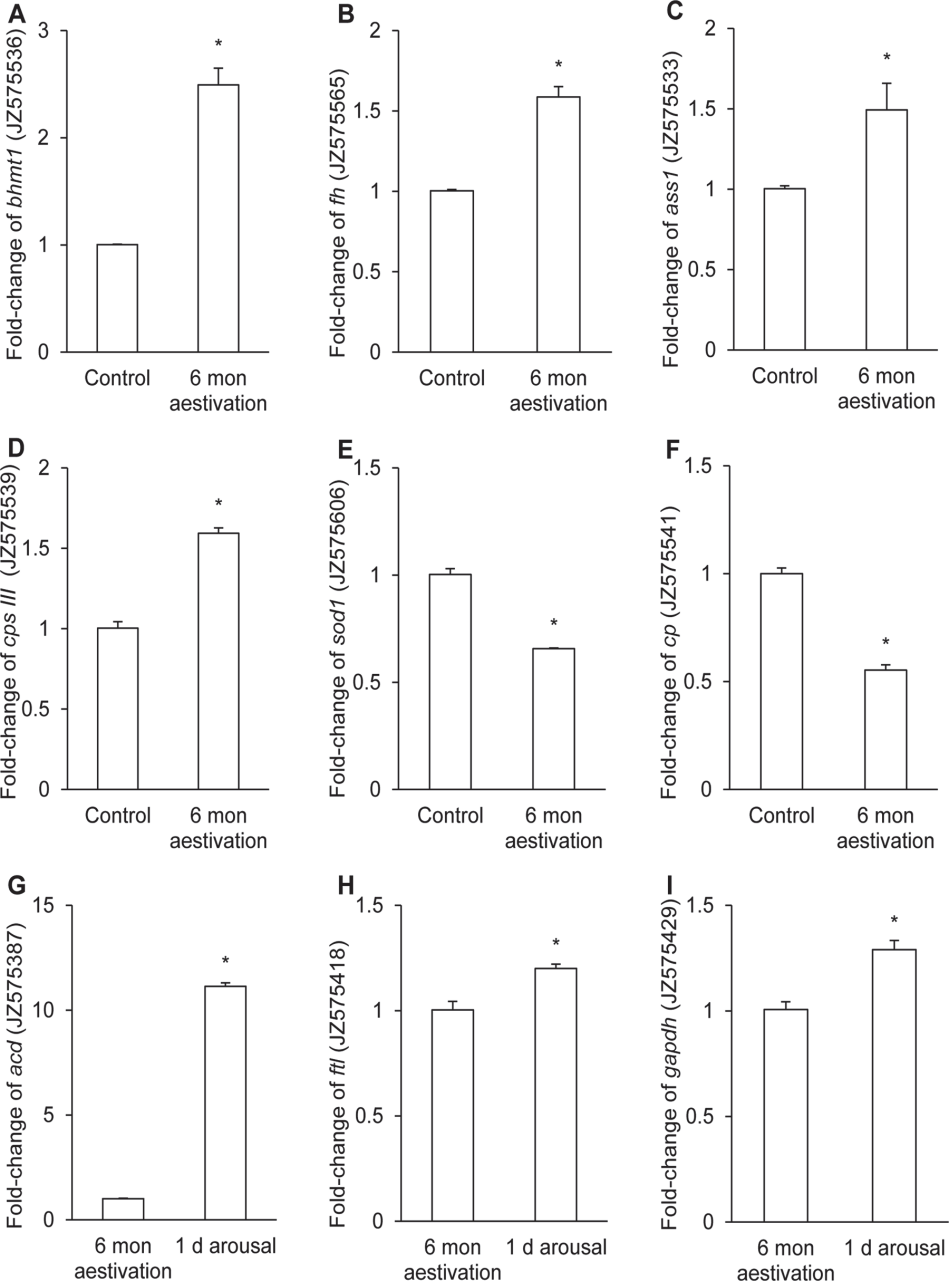
Quantitative RT-PCR results of selected genes that were differentially expressed in the SSH libraries. Relative quantification of mRNA expression (fold change) of (A) *betaine-homocysteine S-methyltransferase 1* (*bhmt1*, JZ575536), (B) *fumarate hydratase* (*fh*, JZ575565), (C) *argininosuccinate synthetase 1* (*ass1*, JZ575533), (D) *carbamoyl-phosphate synthetase III* (*cpsIII*, JZ575539), (E) *superoxide dismutase 1* (*sod1*, JZ575606), (F) *ceruloplasmin* (*cp*, JZ575541), (G) *acyl-CoA desaturase* (*acd*, JZ575387), (H) *ferritin light chain* (*ftl*, JZ575418) and (I) *glyceraldehyde-3-phosphate dehydrogenase* (*gapdh*, JZ575429), using *β-actin* as the reference gene, in the liver of *Protopterus annectens* after 6 months (mon) of aestivation as compared with the freshwater control (A-F), or 1 day (d) of arousal from 6 mon aestivation as compared with fish aestivated for 6 mon (G-I). Results represent mean + S.E.M. (*N* = 6). *Significantly different from the corresponding control (*P*<0.05).

Relative quantification of mRNA expression levels of selected genes were performed using qPCR to verify the up- or down-regulated of selected genes. In agreement with the SSH results of the forward library, there were significant increases in the mRNA expression levels of *bhmt1*, *fh*, *ass1*, *cps III* in the liver of *P*. *annectens* after 6 months of aestivation (Fig. 1A-D). In addition, there were significant decreases in the mRNA expression levels of *sod1* and *cp* in corroboration of the SSH results (Fig. 1E and F).

### SSH libraries from liver of *P*. *annectens* after 1 day of arousal from 6 months of aestivation (with 6 months of aestivation as the driver)

Similarly, forward (Table 4) and reverse (Table 5) libraries were constructed to reflect the genes that were up- and down-regulated, respectively, in the liver of *P*. *annectens* after 1 day of arousal from 6 months of aestivation. A total of 143 genes were identified from these subtraction libraries, in which 76 genes were up-regulated (Table 4) and 67 genes were down-regulated (Table 5). Out of these 1000 sequences obtained, 391 were unidentified and they could again be genes that are yet to be characterized in *P*. *annectens*. *Fructose-bisphosphate aldolase B* (*aldob*) and some genes related to ribosomal proteins appeared in both forward and reverse subtraction libraries, indicating that they could be false positives or encoding for different isoforms of the same protein.

**Table 4:** Known transcripts found in the forward library (up-regulation) obtained by suppression subtractive hybridization PCR from the liver of *Protopterus annectens* after 1 day of arousal from 6 months of aestivation with fish aestivated for 6 months in air as the reference for comparison.

Group and Gene	Gene symbol	*P*. *annectens* accession no.	Homolog species	E-value	No of clones	Biological processes
**Nitrogen metabolism**						
*argininosuccinate synthetase 1*	*ass1*	JZ575395	*Xenopus laevis*	3E-45	7	Arginine biosynthetic process
**Carbohydrate metabolism**						
*glyceraldehyde-3-phosphate dehydrogenase*	*gapdh*	JZ575429	*Xenopus (Silurana) tropicalis*	9E-34	4	Glycolysis
*fructose-bisphosphate aldolase B fragment 1*	*aldob*	JZ575422	*Protopterus annectens*	4E-57	4	Glycolysis
**Lipid metabolism**						
*acyl-CoA desaturase*	*acd*	JZ575387	*Salmo salar*	2E-71	11	Fatty acid biosynthetic process, positive regulation of cholesterol esterification
*desaturase 2*	*fads2*	JZ575411	*Cyprinus carpio*	6E-55	5	Lipid biosynthetic process
*fatty acid-binding protein*	*fabp*	JZ575416	*Platichthys flesus*	2E-05	4	Transport
*stearoyl-CoA desaturase*	*scd*	JZ575507	*Ictalurus punctatus*	9E-35	1	Lipid biosynthetic process
**Amino acid, polyamine and nucleotide metabolism**						
*alanine-glyoxylate aminotransferase*	*agxt*	JZ575390	*Xenopus (Silurana) tropicalis*	6E-65	2	Oxalic acid secretion, glyoxylate metabolic process
*inter-alpha (globulin) inhibitor H3*	*itih3*	JZ575437	*Danio rerio*	9E-09	2	Hyaluronan metabolic process
*inter-alpha trypsin inhibitor*, *heavy chain 2*	*itih2*	JZ575438	*Xenopus laevis*	9E-10	4	Hyaluronan metabolic process
*fumarylacetoacetate hydrolase*	*fah*	JZ575425	*Xenopus laevis*	2E-60	1	Aromatic amino acid family metabolic process
**ATP synthesis**						
*ATP synthase*, *H* ^*+*^ *transporting*, *mitochondrial F*_*0*_ *complex*, *subunit G*	*atp5l*	JZ575396	*Xenopus (Silurana) tropicalis*	4E-36	2	ATP biosynthetic process, ATP synthesis coupled proton transport
*ATP synthase*, *H* ^*+*^ *transporting*, *mitochondrial F*_*1*_ *complex*, *beta polypeptide*	*atp5b*	JZ575397	*Xenopus (Silurana) tropicalis*	6E-84	2	ATP biosynthetic process, proton transport
**Blood coagulation**						
*coagulation factor II*	*f2*	JZ575404	*Xenopus laevis*	2E-37	1	Blood coagulation, platelet activation
**Iron metabolism and transport**						
*ferritin light chain*	*ftl*	JZ575418	*Xenopus (Silurana) tropicalis*	3E-90	27	Cellular iron ion homeostasis, iron ion transport
*ferritin*, *middle subunit*	*frim*	JZ575419	Oncorhynchus mykiss	9E-51	1	Iron ion transport
*transferrin-a*	*tfa*	JZ575511	*Xenopus laevis*	7E-23	2	Cellular iron ion homeostasis
**Protein synthesis, transport and folding**						
*eif4e-binding protein 3*	*eif4ebp3*	JZ575412	*Danio rerio*	6E-27	3	Translational initiation
*eukaryotic translation elongation factor 1 alpha 1*	*eef1a1*	JZ575414	*Xenopus laevis*	5E-101	7	Translation
*elongation factor-1*, *delta*, *b*	*eef1db*	JZ575413	*Danio rerio*	9E-09	3	Translational elongation, Translation
*cL41b ribosomal protein L41*	*rpl41*	JZ575403	*Cyprinus carpio*	3E-21	4	Translation
*protein AMBP*	*ambp*	JZ575446	*Xenopus laevis*	9E-06	27	Protein maturation, transport
*ribosomal protein L18*	*rpl18*	JZ575584	*Protopterus dolloi*	3E-129	8	Translation
*ribosomal protein L41*	*rpl41*	JZ575484	*Cyprinus carpio*	2E-21	5	Translation
*ribosomal protein L7a-like fragment 1*	*rpl7a*	JZ575469	*Protopterus dolloi*	7E-105	8	Ribosome biogenesis
*ribosomal protein P2*	*rplp2*	JZ575486	*Ictalurus punctatus*	2E-74	5	Translational elongation
*ribosomal protein S12 fragment 1*	*rps12*	JZ575492	*Xenopus laevis*	5E-36	2	Translation
*ribosomal protein S2 fragment 1*	*rps2*	JZ575487	*Xenopus laevis*	5E-61	2	Translation
*ribosomal protein S7*	*rps7*	JZ575489	*Protopterus dolloi*	0	1	Translation
*sec61 beta subunit*	*sec61b*	JZ575498	*Xenopus (Silurana) tropicalis*	6E-65	2	Protein transport
**Transcription**						
*fusion*, *derived from t(12;16) malignant liposarcoma*	*fus*	JZ575426	*Xenopus laevis*	8E-61	2	Positive regulation of transcription from RNA polymerase II promoter
*non-pou domain containing*, *octamer binding*	*nono*	JZ575458	*Homo sapiens*	9E-11	3	RNA splicing, cellular transcription
*transformer-2 alpha*	*tra2a*	JZ575512	*Xenopus (Silurana) tropicalis*	2E-74	3	RNA splicing
**Oxidation reduction**						
*NADH dehydrogenase (ubiquinone) 1 alpha subcomplex*, *2*	*ndufa2*	JZ575453	*Danio rerio*	7E-37	5	Electron transport chain
*3-hydroxybutyrate dehydrogenase*, *type 1*	*bdh1*	JZ575382	*Danio rerio*	1E-05	5	Oxidation reduction
*cytochrome c oxidase subunit IV isoform 2*	*cox4i2*	JZ575407	*Xenopus (Silurana) tropicalis*	3E-28	2	Oxidation reduction
*cytochrome P450*, *family 3*, *subfamily A*, *polypeptide 7*	*cyp3a7*	JZ575409	*Homo sapiens*	8E-14	1	Oxidation reduction
**Protein degradation**						
*aminopeptidase-like 1*	*npepl1*	JZ575394	*Xenopus laevis*	3E-75	3	Proteolysis
*cathepsin K*	*ctsk*	JZ575402	*Xenopus (Silurana) tropicalis*	8E-36	2	Proteolysis
*matrix metallopeptidase 1 (interstitial collagenase)*	*mmp1*	JZ575448	*Homo sapiens*	1E-10	3	Collagen catabolic process, proteolysis
*proteasome subunit beta type-3*	*psmb3*	JZ575462	*Salmo salar*	7E-14	4	Proteolysis
**Antioxidative stress**						
*glutathione-S-transferase*	*gst*	JZ575428	*Pleuronectes platessa*	6E-27	13	Antioxidant
**Response to stimulus**						
*cold-inducible RNA-binding protein*	*cirbp*	JZ575405	*Salmo salar*	5E-32	6	Response to stress, stress granule assembly
*heat shock cognate 70*.*II protein*	*hsc70*	JZ575430	*Danio rerio*	9E-67	1	Response to stress
**Apoptosis**						
*cytochrome c*, *somatic*	*cycs*	JZ575408	*Xenopus laevis*	9E-46	2	Apoptosis, electron transport chain
*nuclear protein 1 putative*	*nupr1*	JZ575459	*Salmo salar*	7E-09	5	Positive regulation of apoptosis
**Transport**						
*alpha 1 microglobulin*	*iti*	JZ575391	*Xenopus (Silurana) tropicalis*	5E-08	19	Protein maturation, transport
*globin*, *alpha*	*hba*	JZ575427	*Rattus norvegicus*	1E-13	5	Erythrocyte development, oxygen transport
*mitochondrial glutamate carrier 1*	*slc25a22*	JZ575450	*Salmo salar*	3E-15	3	Transmembrane transport
*solute carrier family 25 (mitochondrial carrier; phosphate carrier)*, *member 3*	*slc25a3*	JZ575504	*Xenopus laevis*	4E-108	1	Transmembrane transport
*transthyretin*	*ttr*	JZ575513	*Danio rerio*	9E-06	1	Transport
**Cell structure**						
*actin-related protein 2/3 complex subunit 4*	*arpc4*	JZ575386	*Xenopus laevis*	6E-78	4	Actin filament polymerization
**Others**						
*ATP-binding cassette*, *sub-family E (OABP)*, *member 1*	*abce1*	JZ575398	*Xenopus laevis*	7E-81	2	Unclassified
*b fibrinopeptide*	*fgb*	JZ575399	*Xenopus laevis*	4E-18	1	Unclassified
*deiodinase type III*	*dio3*	JZ575410	*Neoceratodus forsteri*	2E-26	3	Thyroid hormone catabolic process, hormone biosynthetic process
*histone H1*.*0*	*h1f0*	JZ575435	*Salmo salar*	8E-17	2	Nucleosome assembly
*inter-alpha-inhibitor H2 chain*	*itih2*	JZ575439	*Xenopus laevis*	9E-16	6	Unclassified
*kh domain-containing transcription factor B3 fragment 1*	*igf2bp3-b*	JZ575441	*Xenopus laevis*	6E-09	2	Unclassified
*kh domain-containing transcription factor B3 fragment 2*	*igf2bp3-b*	JZ575442	*Xenopus laevis*	6E-09	2	Unclassified
*kunitz-like protease inhibitor*	*spint1*	JZ575443	*Perca flavescens*	3E-78	3	Unclassified
*lipoprotein*, *Lp(a)*	*lpa*	JZ575445	*Xenopus laevis*	3E-33	2	Unclassified
*mitochondrial Ca* ^*2+*^-*dependent solute carrier 25*	*slc25a25*	JZ575449	*Xenopus laevis*	3E-126	5	Unclassified
*myosin regulatory light chain 2*, *smooth muscle major isoform*	*myl2*	JZ575451	*Rana catesbeiana*	1E-53	1	Unclassified
*prothymosin*, *alpha*	*ptma*	JZ575464	*Xenopus (Silurana) tropicalis*	5E-12	1	Unclassified
*ribosomal protein 5S-like protein*	*rna5s*	JZ575582	*Prionace glauca*	2E-58	6	Unclassified
*ribosomal protein L26 fragment 1*	*rpl26*	JZ575476	*Xenopus (Silurana) tropicalis*	2E-45	1	Unclassified
*run domain-containing protein 1*	*rundc1*	JZ575497	*Salmo salar*	5E-15	2	Unclassified
*saxiphilin precursor*	*sax*	JZ575597	*Rana catesbeiana*	6E-11	1	Unclassified
*snrnp-associated protein*	*snrpb*	JZ575502	*Danio rerio*	8E-74	5	Unclassified
*solute carrier family 3*, *member 1*	*slc3a1*	JZ575503	*Xenopus (Silurana) tropicalis*	8E-17	8	Unclassified
*splicing factor*, *arginine/serine-rich 1*, *like*	*srsf1*	JZ575506	*Danio rerio*	8E-17	3	Unclassified
*tyrosine 3-monooxygenase / tryptophan 5-monooxygenase activation protein*, *epsilon polypeptide*	*ywhae*	JZ575515	*Xenopus (Silurana) tropicalis*	8E-60	6	Protein targeting
*hemopexin-like*	*hpx*	JZ575434	*Maylandia zebra*	3E-28	5	Unclassified
*hemopexin transcript variant 2*	*hpx*	JZ575433	*Xenopus (Silurana) tropicalis*	1E-31	19	Unclassified
*warm temperature acclimation protein 65 KDa-2*	*wap65-2*	JZ575518	*Ictalurus punctatus*	9E-15	36	Unclassified
*Y box binding protein 1 isoform 2*	*ybx1*	JZ575519	*Xenopus laevis*	6E-52	1	Unclassified

**Table 5:** Known transcripts found in the reverse SSH library (down-regulation) obtained by suppression subtractive hybridization PCR from the liver of *Protopterus annectens* after 1 day of arousal from 6 months of aestivation with fish aestivated for 6 months in air as the reference for comparison.

Group and Gene	Gene symbol	*P*. *annectens* accession no.	Homolog species	E-value	No of clones	Biological processes
**Carbohydrate metabolism**						
*fructose-bisphosphate aldolase B fragment 2*	*aldob*	JZ575423	*Protopterus annectens*	9E-145	7	Glycolysis
*plasma alpha-L-fucosidase precursor putative*	*fuca*	JZ575460	*Salmo salar*	7E-10	2	Carbohydrate metabolic process, fucose metabolic process
**Protein synthesis, transport and folding**						
*60S ribosomal protein L32*	*rpl32*	JZ575383	*Xenopus (Silurana) tropicalis*	2E-92	1	Translation
*60S ribosomal protein L35*	*rpl35*	JZ575384	*Xenopus (Silurana) tropicalis*	4E-83	7	Translation
*60S ribosomal protein L36*	*rpl36*	JZ575385	*Xenopus laevis*	8E-60	2	Translation
*eukaryotic translation elongation factor 2*	*eef2*	JZ575415	*Xenopus (Silurana) tropicalis*	9E-27	6	Translation
*Finkel-Biskis-Reilly murine sarcoma virus (FBR-MuSV) ubiquitously expressed (fox derived)*	*fau*	JZ575421	*Xenopus (Silurana) tropicalis*	3E-39	3	Translation
*ribosomal protein L12*	*rpl12*	JZ575472	*Salmo salar*	6E-04	4	Translation
*ribosomal protein L17*	*rpl17*	JZ575473	*Protopterus dolloi*	3E-136	3	Translation
*ribosomal protein L19*	*rpl19*	JZ575474	*Protopterus dolloi*	2E-99	3	Translation
*ribosomal protein L23*	*rpl23*	JZ575475	*Xenopus (Silurana) tropicalis*	1E-110	2	Translation
*ribosomal protein L27a*	*rpl27a*	JZ575478	*Xenopus laevis*	2E-80	2	Translation
*ribosomal protein L3*	*rpl3*	JZ575467	*Xenopus (Silurana) tropicalis*	2E-118	9	Translation
*ribosomal protein L30*	*rpl30*	JZ575479	*Xenopus laevis*	1E-123	5	Translation
*ribosomal protein L32*	*rpl32*	JZ575480	*Xenopus (Silurana) tropicalis*	4E-100	1	Translation
*ribosomal protein L34*	*rpl34*	JZ575481	*Xenopus laevis*	4E-82	2	Translation
*ribosomal protein L36*	*rpl36*	JZ575482	*Xenopus laevis*	2E-77	10	Translation
*ribosomal protein L38*	*rpl38*	JZ575483	*Danio rerio*	1E-50	1	Translation
*ribosomal protein L6*	*rpl6*	JZ575468	*Salmo salar*	3E-10	1	Translation
*ribosomal protein L7a-like fragment 2*	*rpl7a*	JZ575470	*Protopterus dolloi*	2E-74	8	Ribosome biogenesis
*ribosomal protein L9*	*rpl9*	JZ575471	*Xenopus laevis*	2E-67	4	Translation
*ribosomal protein Large P0*	*rplp0*	JZ575485	*Protopterus dolloi*	0	4	Translational elongation
*ribosomal protein S11*	*rps11*	JZ575491	*Xenopus (Silurana) tropicalis*	2E-113	5	Translation
*ribosomal protein S12 fragment 2*	*rps12*	JZ575493	*Xenopus laevis*	2E-41	1	Translation
*ribosomal protein S15*	*rps15*	JZ575494	*Xenopus (Silurana) tropicalis*	6E-112	4	Translation
*ribosomal protein S2 fragment 2*	*rps2*	JZ575488	*Danio rerio*	5E-70	8	Translation
*ribosomal protein S24*	*rps24*	JZ575495	*Xenopus (Silurana) tropicalis*	4E-90	2	Translation
*ribosomal protein S27a*	*rps27a*	JZ575496	*Xenopus (Silurana) tropicalis*	1E-112	1	Translation
*ribosomal protein S9*	*rps9*	JZ575490	*Protopterus dolloi*	0	10	Translation
**Signaling**						
*alpha fetoprotein*	*afp*	JZ575392	*Mus musculus*	4E-27	13	SMAD protein signal transduction, transport
*rho GTPase activating protein 29*	*arhgap29*	JZ575466	*Danio rerio*	7E-09	2	Signal transduction
*secretogranin II*	*scg2*	JZ575499	*Xenopus laevis*	8E-09	1	MAPKKK cascade, angiogenesis
**Structure**						
*thymosin-beta 4*	*tmsb4*	JZ575510	*Amolops loloensis*	7E-24	5	Actin cytoskeleton organization, sequestering of actin monomers
*tubulin*, *beta 2C*	*tubb2c*	JZ575514	*Xenopus (Silurana) tropicalis*	3E-10	2	Protein polymerization, microtubule-based process
**Iron metabolism and transport**						
*alpha globin chain*	*hba*	JZ575393	*Rattus norvegicus*	4E-15	15	Oxygen transport
*ferritin heavy chain*	*fth*	JZ575417	*Bufo gargarizans*	3E-84	1	cellular iron ion homeostasis, iron ion Transport
*hemoglobin alpha 3 subunit*	*hba3*	JZ575432	*Xenopus (Silurana) tropicalis*	3E-07	1	Oxygen transport
*transferrin*	*tf*	JZ575610	*Salmo marmoratus*	2E-12	22	Iron ion transport, cellular iron ion Homeostasis
**Protein degradation**						
*carboxypeptidase B2*	*cpb2*	JZ575401	*Xenopus (Silurana) tropicalis*	5E-26	5	Proteolysis
*hyaluronan binding protein 2*	*habp2*	JZ575436	*Danio rerio*	3E-16	1	Proteolysis
**Transcription**						
*basic leucine zipper and W2 domains 1*	*bzw1*	JZ575400	*Xenopus (Silurana) tropicalis*	7E-73	2	Regulation of cellular transcription
*nascent polypeptide-associated complex alpha subunit isoform b*	*naca*	JZ575455	*Xenopus (Silurana) tropicalis*	2E-56	2	Cellular transcription
**Oxidation reduction**						
*NADH dehydrogenase 1 beta subcomplex subunit 8*, *mitochondrial precursor putative*	*ndufb8*	JZ575454	*Esox lucius*	1E-57	1	Electron transport chain
*urate oxidase*	*uox*	JZ575516	*Protopterus annectens*	0	1	Purine base metabolic process, oxidation reduction
**Transport**						
*adaptor-related protein complex 4*, *mu 1 subunit*	*ap4m1*	JZ575388	*Danio rerio*	4E-72	5	Intracellular protein transport
*retinol binding protein*	*rbp*	JZ575465	*Cyprinus carpio*	3E-43	1	Retinoic acid metabolic process, transport
*serum albumin*	*alb*	JZ575602	*Ornithorhynchus anatinus*	6E-50	1	Transport
*solute carrier family 41*, *member 2*	*slc41a2*	JZ575505	*Xenopus (Silurana) tropicalis*	4E-06	4	Ion transport
**Others**						
*alanine*:*glyoxylate aminotransferase-like*	*agxt*	JZ575389	*Xenopus laevis*	7E-48	3	Unclassified
*cyclophilin A*	*ppia*	JZ575406	*Xenopus laevis*	9E-54	2	Protein folding
*fetuin B*	*fetub*	JZ575420	*Xenopus (Silurana) tropicalis*	6E-23	15	Unclassified
*fukutin related protein isoform 2*	*fkrp*	JZ575424	*Xenopus (Silurana) tropicalis*	3E-08	12	Glycoprotein biosynthetic process
*heat shock protein 20*	*hspb6*	JZ575431	*Ostertagia ostertagi*	6E-24	1	Response to stress, response to heat
*isopentenyl-diphosphate delta isomerase 1*	*idi1*	JZ575440	*Danio rerio*	1E-04	1	Lipid biosynthetic process
*lem domain containing 3*	*lemd3*	JZ575444	*Danio rerio*	1E-11	3	Unclassified
*macrophage migration inhibitory factor*	*mif*	JZ575447	*Xenopus laevis*	4E-11	3	Regulation of cell proliferation, innate immune response
*myotubularin*	*mtm1*	JZ575452	*Xenopus laevis*	2E-14	1	Muscle homeostasis, dephosphorylation
*ndrg2 protein*	*ndrg2*	JZ575456	*Xenopus (Silurana) tropicalis*	1E-16	1	Cell differentiation
*nk2 transcription factor related 2a*	*nkx2*.*2a*	JZ575457	*Danio rerio*	3E-08	1	Unclassified
*plasminogen activator inhibitor 1 RNA-binding protein*	*serpine1*	JZ575461	*Salmo salar*	1E-32	1	Unclassified
*protein tyrosine phosphatase*, *receptor type*, *U*	*ptpru*	JZ575463	*Xenopus (Silurana) tropicalis*	2E-06	2	Protein amino acid dephosphorylation
*ribosomal protein L26 fragment 2*	*rpl26*	JZ575477	*Pelodiscus sinensis*	5E-66	1	Unclassified
*serine protease inhibitor*	*a1at*	JZ575500	*Cyprinus carpio*	4E-08	2	Unclassified
*serine/threonine kinase receptor associated protein*	*strap*	JZ575501	*Danio rerio*	1E-13	2	RNA splicing, mRNA processing
*swi/snk related*, *matrix associated*, *actin dependent regulator of chromatin*, *subfamily a*, *member 4*	*smarca4*	JZ575508	*Danio rerio*	6E-17	1	Unclassified
*tetratricopeptide repeat domain 11*	*ttc11*	JZ575509	*Xenopus laevis*	1E-11	3	Apoptosis
*vitelline membrane outer layer protein 1 homolog precursor putative*	*vmo1*	JZ575517	*Rana catesbeiana*	7E-19	2	Unclassified

As revealed by the forward library (up-regulation), the mRNA expressions of *aldob* and a*ss1*, related to carbohydrate and nitrogen metabolism, respectively, were up-regulated in the liver of *P*. *annectens* after 1 day of arousal from 6 months of aestivation. Some genes involved in lipid metabolism (*acd*, *desaturase 2*, *fatty acid binding protein* and *stearoyl-CoA desaturase*), ATP synthesis and iron metabolism (*ftl*, *ferritin middle subunit* and *transferrin-a*) were also up-regulated (Table 4).

The reverse library (down-regulation) revealed that the down-regulation of expression levels of certain genes related to carbohydrate metabolism (*aldob* and *plasma alpha-L-fucosidase precursor putative*) in the liver of *P*. *annectens* after 1 day of arousal from 6 months of aestivation. The mRNA expression levels of some genes related to protein synthesis, signaling and iron metabolism (*alpha globin chain*, *ferritin heavy chain* and *transferrin*) were also down-regulated (Table 5).

In support of the SSH results, there were significant increases in the mRNA expression levels of *acd*, *ftl* and *gapdh* in the liver of *P*. *annectens* after 1 day of arousal from 6 months of aestivation as confirmed by qPCR (Fig. 1G-I).

## Discussion

### Maintenance phase: up-regulation of ornithine-urea cycle (OUC) capacity

African lungfishes are ureogenic and they possess a full complement of OUC enzymes including CpsIII in their livers [8,18,19]. During the maintenance phase of aestivation, ammonia released through amino acid catabolism must be detoxified because its excretion would have been completely impeded during desiccation [12]. By synthesizing and accumulating urea, which is less toxic, *P*. *annectens* can carry out protein catabolism for a longer period without being intoxicated by ammonia [12]. Therefore, there is a need to increase the urea-synthesizing capacity during the maintenance phase of aestivation. Indeed, there were increases in mRNA expression levels of OUC enzymes, particularly *ass1* and *cpsIII*, in the liver of *P*. *annectens* after 6 months of aestivation (Table 1). There was also a significant increase in the expression level of *fh*. Fh catalyzes the reversible conversion between fumarate and malate and is believed to play an important role in the tricarboxylic acid cycle [20]. It can also be involved in nitrogen metabolism as it could regulate the fumarate levels produced by the OUC [20].

### Maintenance phase: up-regulation of *bhmt1*

BHMT is a cytosolic zinc metalloprotein belonging to the family of methyltransferases [21]. It catalyzes the transfer of a methyl group to homocysteine to form methionine [22], and contributes to ~50% of methionine synthesis in liver [23]. In human, defects in methionine and cysteine metabolism in the liver lead to increased homocysteine concentration in the plasma, i.e. hyperhomocysteinemia, which is associated with vascular diseases [24,25], birth defects such as spina bifida [26], and neurodegenerative diseases such as Alzheimer’s disease [27]. When accumulated abnormally in tissues and organs, homocysteine can produce multiple deleterious changes simultaneously [28], leading to multi-organ failure involving the brain, kidney, heart, vascular system and/or musculoskeletal system [29–32]. Hence, it is highly probable that *bhmt1*/Bhmt1 expressions were up-regulated in the liver of *P*. *annectens* to reduce the hepatic homocysteine concentration during the maintenance phase of aestivation as suggested by Ong et al. [33].

### Maintenance phase: down-regulation of genes related to blood coagulation

As the heart rate of African lungfish, *P*. *aethiopicus*, drops from 22–30 beats min^-1^ before aestivation to 12–17 beats min^-1^ by the end of 1–1.5 months in the mud [34], it is probable that a severe decrease in the rate of blood flow would have occurred. Thus, any mechanism that can prevent the formation of a thrombosis when the fish is inactive during aestivation would be of considerable survival value. Indeed, several genes related to blood coagulation, which included *fibrinogen* (7 clones), *apolipoprotein H* (8 clones) and *serine proteinase inhibitor clade C (antithrombin) member 1* (*serpinc1*; 3 clones) were down-regulated in the liver of fish after 6 months of aestivation (Table 3) and this could signify a decrease in the tendency of blood clot formation.

### Maintenance phase: down-regulation of *sod1*

SOD is an antioxidant enzyme that catalyzes the dismutation of two O_2_^•-^ to H_2_O_2_, and therefore plays a central role in antioxidation. An adaptive response against oxidative stress is often marked by the increased production of intracellular antioxidant enzymes such as SOD, catalase, glutathione peroxidase and glutathione reductase to protect the macromolecules from the stress-induced damage. It was suggested that up-regulation of intracellular antioxidant enzymes during aestivation and hibernation protects against stress-related cellular injury [35,36]. However, the down-regulation in the mRNA expression of *sod1* in the liver of *P*. *annectens* after 6 months of aestivation (Table 3) suggests that other antioxidant enzymes such as Bhmt1, glutathione-S-transferase, glutathione reductase, glutathione peroxidase or catalase may be involved and their activities would be sufficient to counteract the oxidative stress. Also, these results could be indicative of a decrease in ROS production during the maintenance phase of aestivation due to a slower metabolic rate, including the rate of nitrogen metabolism.

### Maintenance phase: down-regulation of genes related to complement fixation

The complement system mediates a chain reaction of proteolysis and assembly of protein complexes that results in the elimination of invading microorganisms [37,38]. Three activation pathways (the classical, lectin and alternative pathways) and a lytic pathway regulate these events. *Protopterus annectens* utilizes lectin pathway for protection against pathogens during the induction phase of aestivation [13]. However, our results showed that many genes related to complement fixation appeared in the reverse library. These included the *complement C3 precursor alpha chain* (11 clones), *complement component 4 binding protein alpha* (3 clones) and *CD46 antigen complement regulatory protein* (2 clones), and seven others (Table 3). Hence, *P*. *annectens* might down-regulate the classical complement fixation pathway during the maintenance phase of aestivation, possibly because of three reasons. Firstly, the dried mucus cocoon was already well formed, which conferred the aestivating lungfish a certain degree of protection against external pathogens. Secondly, tissue reconstruction would have subsided after the induction phase, and there could be minimal tissue inflammation during the prolonged maintenance phase. Thirdly, it was important to conserve the limited energy resources, and it would be energetically demanding to sustain the increased expression of genes involved in complement fixation during the maintenance phase of aestivation.

### Maintenance phase: down-regulation of warm-temperature-acclimation-related 65 kDa protein and hemopexin

The plasma glycoprotein warm-temperature-acclimation-related protein (Wap65) was first identified in the goldfish *Carassius auratus* [39] and the cDNA showed a homology of 31% to rat hemopexin, a serum glycoprotein that transports heme to liver parenchymal cells [40]. Hemopexins in mammals are mainly synthesized in liver and are responsible for the transportation of heme resulting from hemolysis to the liver. Therefore, the down-regulation of the *wap65* and *hemopexin* in the liver of *P*. *annectens* (Table 3) suggested that hemolysis might be suppressed during the maintenance phase of aestivation. There are also indications that the Wap65 can be involved in immune responses in the Channel catfish *Ictalurus punctatus* [41]. Hence, its down-regulation suggested that a decrease in immune response might have occurred in the liver of *P*. *annectens* during the maintenance phase of aestivation.

### Maintenance phase: down-regulation of genes related to iron metabolism

Iron is involved in many cellular metabolic pathways and enzymatic reactions, but it is toxic when in excess [42–44]. Transferrin is one of the major serum proteins, which is synthesized mainly in liver and plays a crucial role in iron metabolism. Under normal conditions, most of the iron in the plasma is bound to transferrin, and iron-transferrin complexes enter the cells via a transferrin receptor-mediated endocytic pathway. Transferrin also has a close relationship with the immune system. It binds to iron, creating an environment with low levels of iron, where few microorganisms can survive and prosper [45]. On the other hand, ferritin is the main iron storage protein in both eukaryotes and prokaryotes; it keeps iron in a soluble and non-toxic form [43,46,47]. Also, up-regulation of ferritin has been observed in oxidative stress [48] and inflammatory conditions in human [49–51]. *Transferrin* and *ferritin* mRNA expression levels are up-regulated in *P*. *annectens* during the induction phase of aestivation [13], probably due to oxidative stress and inflammation arisen through tissue reconstruction, and/or a high turnover rate of free and bound iron resulting from increased production of certain types of hemoglobins or hemoglobin in general. By contrast, our results indicated that there could be a decrease in the capacity of iron metabolism and transport in *P*. *annectens* during the maintenance phase of aestivation as *transferrin* (14 clones) and *hemopexin* (3 clones) appeared in the reverse library. This correlated well with the aestivation process as a prolonged torpor state would theoretically lead to a lower rate of ROS production, and stabilized expression of hemoglobin genes.

### Maintenance phase: down-regulation of genes related to copper metabolism

Ceruloplasmin (CP) is crucial in the oxidation of Fe^2+^ to Fe^3+^, which enables the binding of iron to transferrin, facilitating the mobilization of iron in the body. It also represents a tightly bound pool of copper that accounts for >90% of the total plasma copper in most species [52,53]. CP synthesis and/or secretion can be altered by inflammation, hormones, and copper. Plasma concentrations of acute-phase globulins, including CP, increase with tissue injury, localized acute inflammation, and chronic inflammatory diseases [54]. The mRNA expression level of *cp* was up-regulated in the liver of *P*. *annectens* during the induction phase of aestivation [13]. However, our results revealed that 6 months of aestivation led to a down-regulation of *cp* mRNA expression in the liver of *P*. *annectens*. This suggested that tissue degradation or inflammation may be limited during the maintenance phase of aestivation due to a profound decrease in metabolic activity. Consequently, there was no longer a need to up-regulate expression level of *cp*.

### Maintenance phase: up- or down-regulation of protein synthesis?

Twelve genes related to protein synthesis, transport and folding appeared in the reverse library of lungfish undergoing 6 months of aestivation in air (Table 3). The down-regulation of genes related to protein synthesis such as eukaryotic translation initiation factors and other ribosomal proteins is a consistent phenomenon in metabolic rate reduction. Suppression of protein synthesis during aestivation would help the animal to conserve energy and enhance its survival. However, 10 types of ribosomal proteins appeared in the forward library indicating up-regulation of mRNA expressions of these genes in the liver of *P*. *annectens* after 6 months of aestivation (Table 2). Taken altogether, these results indicate that the capacity of protein synthesis was not suppressed completely during the prolonged phase of aestivation. This could be an important strategy since the aestivating lungfish would have to maintain the protein synthesis machinery in preparation for arousal from aestivation when water becomes available.

### Arousal phase: up-regulation of *ass1* expression and amino acid metabolism

After 1 day of arousal from 6 months of aestivation, *ass1* still appeared in the forward library (Table 4), indicating that there was a further increase in the mRNA expression of *ass1* in the liver. Since *cpsIII* and *fh* could not be found in the reverse library (Table 5), and their mRNA expressions were already up-regulated during the maintenance phase of aestivation, it can be deduced that their increased mRNA expressions were sustained into the arousal phase.

Upon arousal, the fish has to reconstruct cells and tissues that have been modified during the induction phase and repair damages that have occurred during the maintenance phase of aestivation. Such structural changes would require increased syntheses of certain proteins, and since refeeding would not occur until 7–10 days after arousal, it would imply the mobilization of amino acids of endogenous origin [12]. Both substrate and energy are needed for repair and regeneration. Our results indicate that endogenous amino acids could serve such purposes during arousal. Indeed, there could be increases in the capacity of protein turnover, the electron transport system, lipid biosynthesis and iron metabolism in *P*. *annectens* after 1 day of arousal from 6 months of aestivation. The energy that supports these activities could be derived from increased amino acid (and perhaps also carbohydrate) catabolism during this period. The ammonia released through increased amino acid catabolism had to be detoxified to urea through the hepatic OUC. Therefore, it can be understood why there were significant increases in the urea-synthesizing capacity upon arousal from aestivation.

Besides being involved in urea synthesis, arginine produced by Ass also acts as a substrate for nitric oxide (NO) production in the liver, where NO is involved in liver regeneration [55] and protection of the liver from ischaemia–reperfusion injury [56]. Indeed, Chng et al [57] had shown that the arginine and NOx concentrations decreased and increased, respectively, in the liver of *P*. *annectens* after 6 months of aestivation and after 3 days of arousal from aestivation, supporting the proposition that arginine synthesized through Ass could be used for increased NO production, especially during arousal.

### Arousal phase: up-regulation of carbohydrate metabolism?

Compared with the maintenance phase, 1 day of arousal led to increases in mRNA expressions of *gapdh* and *aldob*, and a decrease in the expression of another isoform of *aldob*. Although Gapdh does not catalyse a flux generating step (unlike hexokinase, glycogen phosphorylase, and pyruvate kinase) or act as a regulatory enzyme (unlike phosphofructokinase) in the glycolytic pathway, it involves an oxidation-reduction reaction, and our results could indicate a tendency towards an up-regulation of carbohydrate metabolism in the liver of *P*. *annectens* during the arousal phase of aestivation. Frick et al. [58] reported that *P*. *dolloi* conserved the glycogen pool during the maintenance phase of aestivation. Naturally, the fish becomes more active after arousal, and there could be an increase in the utilization of glycogen store for energy production during this period before feeding is resumed.

### Arousal phase: up-regulation of genes involved in lipid metabolism and fatty acid transport

Fatty acid binding proteins (FABPs) are intracellular carriers that transport fatty acids through cytoplasm, linking sites of fatty acid import/export (plasma membrane), internal storage (lipid droplets), and oxidation (mitochondria) [59]. Stearoyl-CoA desaturase is a lipogenic enzyme that catalyzes the synthesis of monounsaturated fatty acids [60]. Acyl-CoA desaturase is the terminal component of the liver microsomal stearoyl-CoA desaturase system that utilizes O_2_ and electrons from reduced cytochrome b5 to catalyze the insertion of a double bond into a spectrum of fatty acyl-CoA substrates including palmitoyl-CoA and stearoyl-CoA. The up-regulation of mRNA expressions of *fabps* (4 clones), *stearoyl-CoA desaturase* (1 clone), *desaturase* (5 clones) and *acyl-CoA desaturase* (11 clones) (Table 4) indicate that there could be an increase in fatty acid synthesis and lipid metabolism in the liver of *P*. *annectens* after 1 day of arousal. Tissue regeneration would be an important activity during arousal, and cell proliferation requires increased lipid metabolism to generate biomembranes. It is probable that the energy required to sustain these activities was derived from amino acid catabolism.

### Arousal phase: up-regulation of electron transport system and ATP synthesis?

Conservation of energy is a key feature during the maintenance phase of aestivation to sustain life in adverse environmental condition. Arousal from aestivation marks an increase in the demand for ATP. Indeed, after 1 day of arousal, there were increases in mRNA expressions of *ndufa2* (5 clones), *cytochrome c oxidase subunit IV isoform 2* (2 clones) and two different types of *ATP synthase* (*mitochondrial F*_*o*_ *and F*_*1*_ *complex*; 2 clones each) (Table 4), indicating that mitochondria became more active. It would be essential to maintain mitochondrial redox balance when activities of oxidation-reduction reactions increased in the mitochondrial matrix. The increase in mRNA expression of *3-hydroxybutyrate dehydrogenase type 1* (5 clones) suggested that mitochondrial activities might not be fully supported by an adequate supply of oxygen, and mitochondrial redox balance might have been maintained transiently through hydroxybutyrate formation during this initial phase of arousal.

### Arousal phase: up- or down-regulation of iron metabolism and transport

There could be two reasons for the increases in *transferrin* and *ferritin* expressions in the liver of *P*. *annectens* during arousal. Firstly, it could be a response to increased oxidative stress and inflammation. After arousal, the lungfish would immediately swim to the surface to breathe air. A rapid increase in O_2_ metabolism would lead to increased generation of reactive oxygen species, as the rate of superoxide generation at the mitochondrial level is known to be correlated positively with oxygen tension [61,62]. Furthermore, animals experiencing transient metabolic depression followed by restoration of normal O_2_ uptake also experience oxidative stress; examples consist of hibernating mammals, anoxia-tolerant turtles, freeze-tolerant frogs and molluscs [35,63,64]. Secondly, it could be due to an increase in the turnover of free and bound iron as a result of the increase in synthesis of certain type of hemoglobins and/or hemoglobin in general. Delaney et al. [65] reported that 4 electrophoretically distinct types of hemoglobins (fraction I, II, III and IV) were present in *P*. *aethiopicus*, and there were increases in the amounts of types II and IV hemoglobins during the maintenance phase of aestivation. Hence, it is logical to deduce that changes in hemoglobin types during the induction phase of aestivation must be reverted back to normal during arousal, which could be one of the reasons that led to the up-regulation in mRNA expressions of *transferrin* and *ferritin* in the liver of *P*. *annectens*.

### Arousal phase: up-regulation of *glutathione S-transferase* (*gst*)

GSTs are a major group of detoxification proteins involved in protecting against various reactive chemicals, including chemical carcinogens, secondary metabolites during oxidative stress, and chemotherapeutic agents [66]. They catalyze the reaction of glutathione with electrophilic centers of organic compounds [67]. These glutathione-conjugated compounds are rendered more water-soluble and more readily excreted. Besides, some GSTs have secondary catalytic activities including steroid isomerisation [68] and a selenium-independent peroxidase activity with organic hydroperoxides [69]. The alpha class GST (GSTa) may also function as intracellular transporters of various hydrophobic compounds (which are not substrates of GSTs) like bilirubin, heme, thyroid hormones, bile salts and steroids [70]. The increase in mRNA expression of *gst* in the liver of *P*. *annectens* after 1 day of arousal (Table 4) is indicative of a possible increase in secondary metabolites of oxidative stress and/or transport of heme in the liver. Similarly, increases in activity of Gst have been observed in aestivating snails and snails aroused from aestivation [71].

### Arousal phase: increase in protein turnover

Based on the variety of genes related to protein synthesis, transport and folding in the forward and reverse library, it can be concluded that there was a high rate of protein turnover in the liver of lungfish after 1 day of arousal. It would appear that the machinery (e.g. ribosomal protein L12, L17 and L19) involved in the maintenance of protein structure during the maintenance phase (Table 4) was different from that (e.g. eIF4E-binding protein, eukaryotic translation elongation factor alpha 1 and elongation factor-1, delta b) involved in the regeneration of protein structure during the arousal phase (Table 5).

## Conclusion

Six months of aestivation led to changes in gene expression related to nitrogen metabolism, oxidative defense, blood coagulation, complement fixation, iron and copper metabolism, and protein synthesis in liver of *P*. *annectens*. These results indicate that sustaining a low rate of waste production and conservation of energy store were essential to the maintenance phase of aestivation. On the other hand, there were changes in gene expression related to nitrogen metabolism, lipid metabolism, fatty acid transport, electron transport system, and ATP synthesis in liver of *P*. *annectens* after 1 day of arousal from 6 months of aestivation. It would appear that the freshly aroused fish depended on internal energy store for repair and structural modification. Overall, our results indicate that aestivation cannot be regarded as the result of a general depression of metabolism only, but it involves the complex interplay between up-regulation and down-regulation of diverse cellular activities. Hence, efforts should be made in the future to identify and differentiate molecular, biochemical and physiological phenomena in African lungfishes incidental to each of the three phases (induction, maintenance and arousal) of aestivation.

## References

[1] Smith HW. Observations on the African lungfish, *Protopterus aethiopicus*, and on evolution from water to land environments. Ecology. 1931;12: 164–181.

[2] Janssens PA, Cohen PP. Biosynthesis of urea in the estivating African lungfish and in *Xenopus laevis* under conditions of water shortage. Comp Biochem Physiol. 1968;24: 887–898. 5650496 10.1016/0010-406x(68)90800-1

[3] Janssens PA, Cohen PP. Nitrogen metabolism in the African lungfish. Comp Biochem Physiol. 1968;24: 879–886. 5689864 10.1016/0010-406x(68)90799-8

[4] DeLaney RG, Fishman AP. Analysis of lung ventilation in the aestivating lungfish *Protopterus aethiopicus* . Am J Physiol Regul Integr Comp Physiol. 1977;233: R181–R187. 10.1152/ajpregu.1977.233.5.R181 920828

[5] Fishman AP, Pack AI, Delaney RG, Galante RJ. Estivation in *Protopterus* . J Morpho. 1986;190 Suppl 1: 237–248.

[6] Chew SF, Chan NK, Loong AM, Hiong KC, Tam WL, Ip YK. Nitrogen metabolism in the African lungfish (*Protopterus dolloi*) aestivating in a mucus cocoon on land. J Exp Biol. 2004;207: 777–786. 14747410 10.1242/jeb.00813

[7] Ip YK, Yeo PJ, Loong AM, Hiong KC, Wong WP, Chew SF. The interplay of increased urea synthesis and reduced ammonia production in the African lungfish *Protopterus aethiopicus* during 46 days of aestivation in a mucus cocoon on land. J Exp Zool. 2005;303A: 1054–1065. 10.1002/jez.a.237 16254918

[8] Loong AM, Hiong KC, Lee SML, Wong WP, Chew SF, Ip YK. Ornithine-urea cycle and urea synthesis in African lungfishes, *Protopterus aethiopicus* and *Protopterus annectens*, exposed to terrestrial conditions for 6 days. J Exp Zool. 2005;303A: 354–365. 10.1002/jez.a.147 15828011

[9] Loong AM, Tan JYL, Wong WP, Chew SF, Ip YK. Defense against environmental ammonia toxicity in the African lungfish, *Protopterus aethiopicus*: bimodal breathing, skin ammonia permeability and urea synthesis. Aquat Toxicol. 2007;85: 76–86. 17881067 10.1016/j.aquatox.2007.08.002

[10] Loong AM, Ang SF, Wong WP, Pörtner HO, Bock C, Bridges CR, et al Effects of hypoxia on the energy status and nitrogen metabolism of African lungfish during aestivation in a mucus cocoon. J Comp Physiol B. 2008;178: 853–865. 10.1007/s00360-008-0273-9 18504593

[11] Loong AM, Pang CYM, Hiong KC, Wong WP, Chew SF, Ip YK. Increased urea synthesis and/or suppressed ammonia production in the African lungfish, *Protopterus annectens*: aestivation in air versus aestivation in mud. J Comp Physiol B. 2008;178: 351–363. 18058110 10.1007/s00360-007-0228-6

[12] Ip YK, Chew SF. Nitrogen metabolism and excretion during aestivation In: Navas CA, Carvalho JE, editors. Progress in molecular and subcellular biology Vol 49, *Aestivation*: *Molecular and Physiological Aspects*. Berlin: Springer-Verlag; 2010 pp. 63–94. 10.1007/978-3-642-02421-4_4 20069405

[13] Loong AM, Hiong KC, Wong WP, Chew SF, Ip YK. Differential gene expression in the liver of the African lungfish, *Protopterus annectens*, after 6 days of estivation in air. J Comp Physiol B. 2012;182: 231–245. 10.1007/s00360-011-0613-z 21915614

[14] Whitehead A, Crawford DL. Variation in tissue-specific gene expression among natural populations. Genome Biol. 2005;6: R13 15693942 10.1186/gb-2005-6-2-r13 PMC551533

[15] Hall TA. BioEdit: a user-friendly biological sequence editor and analysis program for Windows 95/98/NT. Nucleic Acids Symp Ser. 1999;41: 95–98.

[16] Altschul SF, Gish W, Miller W, Myers EW, Lipman DJ. Basic local alignment search tool. J Mol Biol. 1990;215: 403–410. 2231712 10.1016/S0022-2836(05)80360-2

[17] Livak KJ, Schmittgen TD. Analysis of relative gene expression data using real-time quantitative PCR and the 2(-Delta Delta C(T) method. Methods. 2001;25: 402–408. 11846609 10.1006/meth.2001.1262

[18] Chew SF, Ong TF, Ho L, Tam WL, Loong AM, Hiong KC, et al Urea synthesis in the African lungfish *Protopterus dolloi*––hepatic carbamoyl phosphate synthetase III and glutamine synthetase are upregulated by 6 days of aerial exposure. J Exp Biol. 2003;206: 3615–3624. 12966053 10.1242/jeb.00619

[19] Loong AM, Chng YR, Chew SF, Wong WP, Ip YK. Molecular characterization and mRNA expression of carbamoyl phosphate synthetase III in the liver of the African lungfish, *Protopterus annectens*, during aestivation or exposure to ammonia. J Comp Physiol B. 2012;182: 367–379. 10.1007/s00360-011-0626-7 22038021

[20] Yogev O, Yogev O, Singer E, Shaulian E, Goldberg M, Fox TD, et al Fumarase: a mitochondrial metabolic enzyme and a cytosolic/nuclear component of the DNA damage response. PLoS Biol. 2010;8:e1000328 10.1371/journal.pbio.1000328 20231875 PMC2834712

[21] Millian NS, Garrow TA. Human betaine-homocysteine methyltransferase is a zinc metalloenzyme. Arch Biochem Biophys. 1998;356: 93–98. 9681996 10.1006/abbi.1998.0757

[22] Finkelstein JD, Kyle W, Harris BJ. Methionine metabolism in mammals. Regulation of homocysteine methyltransferases in rat tissue. Arch Biochem Biophys. 1971;146: 84–92. 5144037 10.1016/s0003-9861(71)80044-9

[23] Finkelstein JD, Martin JJ. Methionine metabolism in mammals. Distribution of homocysteine between competing pathways. J Biol Chem. 1984;259: 9508–9513. 6746658

[24] Refsum H, Ueland PM, Nygard O, Vollset SF. Homocysteine and cardiovascular disease. Annu Rev Med. 1998;49: 31–62. 9509248 10.1146/annurev.med.49.1.31

[25] Welch GN, Loscalzo J. Homocysteine and atherothrombosis. N Engl J Med. 1998;338: 1042–1050. 9535670 10.1056/NEJM199804093381507

[26] Steegers-Theunissen RP, Boers GH, Trijbels FJ, Eskes TK. Neural-tube defects and derangement of homocysteine metabolism. N Engl J Med. 1991;324: 199–200. 1984202 10.1056/NEJM199101173240315

[27] Miller JW. Homocysteine and Alzheimer's disease. Nutr Rev. 1999;57: 126–129. 10228350 10.1111/j.1753-4887.1999.tb06936.x

[28] Veeranki S, Tyagi SC. Defective homocysteine metabolism: Potential implications for skeletal muscle malfunction. Int J Mol Sci. 2013;14: 15074–15091. 10.3390/ijms140715074 23873298 PMC3742288

[29] Miller A, Mujumdar V, Shek E, Guillot J, Angelo M, Palmer L, et al Hyperhomocyst(e)inemia induces multiorgan damage. Heart Vessels. 2000;15: 135–143. 11289502 10.1007/s003800070030

[30] Maron BA, Loscalzo J. The treatment of hyperhomocysteinemia. Annu Rev Med. 2009;60: 39–54. 10.1146/annurev.med.60.041807.123308 18729731 PMC2716415

[31] Schalinske KL, Smazal AL. Homocysteine imbalance: A pathological metabolic marker. Adv Nutr. 2012;3: 755–762. 10.3945/an.112.002758 23153729 PMC3648699

[32] Kalani A, Kamat PK, Tyagi SC, Tyagi N. Synergy of homocysteine, microRNA, and epigenetics: A novel therapeutic approach for stroke. Mol Neurobiol. 2013;48: 157–168. 10.1007/s12035-013-8421-y 23430482 PMC3695063

[33] Ong JLY, Woo JM, Hiong KC, Ching B, Wong WP, Chew SF, et al Molecular characterization of betaine-homocysteine methyltransferase 1 from the liver, and effects of aestivation on its expressions and homocysteine concentrations in the liver, kidney and muscle, of the African lungfish, *Protopterus annectens* . Comp Biochem Physiol B. 2015;183: 30–41. 25575738 10.1016/j.cbpb.2014.12.007

[34] Delaney RG, Lahiri S, Fishman AP. Aestivation of the African lungfish *Protopterus aethiopicus*: cardiovascular and respiratory functions. J Exp Biol. 1974;61: 111–128. 4411892 10.1242/jeb.61.1.111

[35] Hermes-Lima M, Zenteno-Savín T. Animal response to drastic changes in oxygen availability and physiological oxidative stress. Comp Biochem Physiol C. 2002;133: 537–556. 12458182 10.1016/s1532-0456(02)00080-7

[36] Carey HV, Andrews MT, Martin SL. Mammalian hibernation: cellular and molecular responses to depressed metabolism and low temperature. Physiol Rev. 2003;83: 1153–1181. 14506303 10.1152/physrev.00008.2003

[37] Walport MJ. Complement—first of two parts. N Engl J Med. 2001;344: 1058–1066. 11287977 10.1056/NEJM200104053441406

[38] Walport MJ. Complement—second of two parts. N Engl J Med. 2001;344: 1140–1144. 11297706 10.1056/NEJM200104123441506

[39] Kikuchi K, Yamashita M, Watabe S, Aida K. The warm temperature acclimation-related 65-kDa protein, Wap65, in goldfish and its gene expression. J Biol Chem. 1995;270: 17087–17092. 7615502 10.1074/jbc.270.29.17087

[40] Muller-Eberhard U, Liem HH. Hemopexin: The heme-binding serum glycoprotein In: Allison AC, editor. Structure and function of plasma proteins, vol 1. London: Plenum; 1974 pp 35–53.

[41] Sha Z, Xu P, Takano T, Liu H, Terhune J, Liu Z. The warm temperature acclimation protein Wap65 as an immune response gene: its duplicates are differentially regulated by temperature and bacterial infections. Mol Immunol. 2008;45: 1458–1469. 17920125 10.1016/j.molimm.2007.08.012

[42] Cammack R, Wrigglesworth JM, Baum H. Iron-dependent enzymes in mammalian systems In: Ponka P, Schulman HM, Woodworth RC, editors. Iron transport and storage. Boca Raton: CRC Press; 1990 pp 17–40.

[43] Harrison PM, Arosio P. The ferritins: molecular properties, iron storage function and cellular regulation. Biochim Biophys Acta. 1996;1275: 161–203. 8695634 10.1016/0005-2728(96)00022-9

[44] Linn S. DNA damage by iron and hydrogen peroxide in vitro and in vivo. Drug Metab Rev. 1998;30: 313–326. 9606606 10.3109/03602539808996315

[45] Neves JV, Wilson JM, Rodrigues PNS. Transferrin and ferritin response to bacterial infection: the role of the liver and brain in fish. Dev Comp Immunol. 2009;33: 848–857. 10.1016/j.dci.2009.02.001 19428486

[46] Theil EC. The ferritin family of iron storage proteins. Adv Enzymol Relat Areas Mol Biol. 1990;63: 421–449. 2407067 10.1002/9780470123096.ch7

[47] Chasteen ND. Uptake, storage, and release of iron. Met Ions Biol Syst. 1998;35: 479–514. 9444767

[48] Orino K, Lehman L, Tsuji Y, Ayaki H, Torti SV, Torti FM. Ferritin and the response to oxidative stress. Biochem J. 2001;357: 241–247. 11415455 10.1042/0264-6021:3570241 PMC1221947

[49] Rogers JT, Bridges KR, Durmowicz GP, Glass J, Auron PE, Munro HN. Translational control during the acute phase response. Ferritin synthesis in response to interleukin-1. J Biol Chem. 1990;265: 14572–14578. 1696948

[50] Torti SV, Kwak EL, Miller SC, Miller LL, Ringold GM, Myambo KB, et al The molecular cloning and characterization of murine ferritin heavy chain, a tumor necrosis factor-inducible gene. J Biol Chem. 1988;263: 12638–12644. 3410854

[51] Torti FM, Torti SV. Regulation of ferritin genes and protein. Blood. 2002;99: 3505–3516. 11986201 10.1182/blood.v99.10.3505

[52] Gubler CJ, Lahey ME, Cartwright GE, Wintrobe MM. Studies on copper metabolism. IX. The transportation of copper in blood. J Clin Invest. 1953;32: 405–414. 13052700 10.1172/JCI102752 PMC438356

[53] Henkin R. Metal–albumin–amino acid in interactions: chemical and physiological relationships In: Friedman M, editor. Protein–metal interactions. New York: Plenum; 1974 pp 299–328. 10.1007/978-1-4684-0943-7_15 4611159

[54] Cousins RJ. Absorption, transport, and hepatic metabolism of copper and zinc: special reference to metallothionein and ceruloplasmin. Physiol Rev. 1985;65: 238–309. 3885271 10.1152/physrev.1985.65.2.238

[55] Carnovale CE, Ronco MT. Role of nitric oxide in liver regeneration. Ann Hepatol. 2012;11: 636–647. 22947523

[56] Abu-Amara M, Yang SY, Seifalian A, Davidson B, Fuller B. The nitric oxide pathway-evidence and mechanisms for protection against liver ischaemia reperfusion injury. Liver Int. 2012;32: 531–543. 10.1111/j.1478-3231.2012.02755.x 22316165

[57] Chng YR, Ong JLY, Ching B, Chen XL, Wong WP, Chew SF, et al Molecular characterization of argininosuccinate synthase and argininosuccinate lyase from the liver of the African lungfish *Protopterus annectens*, and their mRNA expression levels in the liver, kidney, brain and skeletal muscle during aestivation. J Comp Physiol B. 2014;184(7): 835–853. 10.1007/s00360-014-0842-z 25034132

[58] Frick NT, Bystriansky JS, Ip YK, Chew SF, Ballantyne JS. Carbohydrate and amino acid metabolism in fasting and aestivating African lungfish (*Protopterus dolloi*). Comp Biochem Physiol. 2008;151: 85–92. 10.1016/j.cbpa.2008.06.003 18593602

[59] Storey KB, Storey JM. Mammalian hibernation: biochemical adaptation and gene expression In: Storey KB, editor. Functional metabolism regulation and adaptation. New York: Wiley; 2004 pp 383–471.

[60] Dobrzyn P, Sampath H, Dobrzyn A, Miyazaki M, Ntambi JM. Loss of stearoyl-CoA desaturase 1 inhibits fatty acid oxidation and increases glucose utilization in the heart. Am J Physiol. 2007;294: E357–E364. 10.1152/ajpendo.00471.2007 18042664

[61] Turrens JF, Freeman BA, Levitt JG, Crapo JD. The effect of hyperoxia on superoxide production by lung submitochondrial particles. Arch Biochem Biophys. 1982;217: 401–410. 6291460 10.1016/0003-9861(82)90518-5

[62] González-Flecha B, Demple B. Metabolic sources of hydrogen peroxide in aerobically growing *Escherichia coli* . J Biol Chem. 1995;270: 13681–13687. 7775420 10.1074/jbc.270.23.13681

[63] Storey KB. Oxidative stress: animal adaptations in nature. Braz J Med Biol Res. 1996;29: 1715–1733. 9222437

[64] Bickler PE, Buck LT. Hypoxia tolerance in reptiles, amphibians, and fishes: life with variable oxygen availability. Annu Rev Physiol. 2007;69: 145–170. 17037980 10.1146/annurev.physiol.69.031905.162529

[65] Delaney RG, Shub G, Fishman AP. Hematologic observations on the aquatic and estivating African lungfish, *Protopterus aethiopicus* . Copeia. 1976;3: 423–434.

[66] Hayes JD, Flanagan JU, Jowsey IR. Glutathione transferases. Annu Rev Pharmacol Toxicol. 2005;45: 51–88. 15822171 10.1146/annurev.pharmtox.45.120403.095857

[67] Ketterer B, Coles B, Meyer DJ. The role of glutathione in detoxication. Environ Health Perspect. 1983;49: 59–69. 6339228 10.1289/ehp.834959 PMC1569131

[68] Benson AM, Talalay P, Keen JH, Jakoby WB. Relationship between the soluble glutathione-dependent delta 5–3-ketosteroid isomerase and the glutathione S-transferases of the liver. Proc Natl Acad Sci USA. 1977;74: 158–162. 264670 10.1073/pnas.74.1.158 PMC393217

[69] Prohaska JR. The glutathione peroxidase activity of glutathione S-transferases. Biochim Biophys Acta. 1980;611: 87–98. 7350921 10.1016/0005-2744(80)90045-5

[70] Mannervik B. Glutathione peroxidase. Methods Enzymol. 1985;113: 490–495. 4088069 10.1016/s0076-6879(85)13063-6

[71] Hermes-Lima M, Storey KB. Antioxidant defenses and metabolic depression in a pulmonate land snail. Am J Physiol. 1995;268: R1386–R1393. 7611513 10.1152/ajpregu.1995.268.6.R1386

